# Preclinical Verification of the Efficacy and Safety of Aqueous Plasma for Ovarian Cancer Therapy

**DOI:** 10.3390/cancers13051141

**Published:** 2021-03-07

**Authors:** Kae Nakamura, Nobuhisa Yoshikawa, Yuko Mizuno, Miwa Ito, Hiromasa Tanaka, Masaaki Mizuno, Shinya Toyokuni, Masaru Hori, Fumitaka Kikkawa, Hiroaki Kajiyama

**Affiliations:** 1 Department of Obstetrics and Gynecology, Nagoya University Graduate School of Medicine, Tsurumai-cho 65, Showa-ku, Nagoya 466-8550, Japan; kikkawaf@med.nagoya-u.ac.jp (F.K.); kajiyama@med.nagoya-u.ac.jp (H.K.); 2 Center for Low-Temperature Plasma Sciences, Nagoya University, Furo-cho, Chikusa-ku, Nagoya 464-8603, Japan; htanaka@plasma.engg.nagoya-u.ac.jp (H.T.); toyokuni@med.nagoya-u.ac.jp (S.T.); hori@nuee.nagoya-u.ac.jp (M.H.); 3 Center for Advanced Medicine and Clinical Research, Nagoya University Hospital, Tsurumai-cho 65, Showa-ku, Nagoya 466-8550, Japan; y-mizuno@med.nagoya-u.ac.jp (Y.M.); miwaito@med.nagoya-u.ac.jp (M.I.); mmizuno@med.nagoya-u.ac.jp (M.M.); 4 Department of Pathology and Biological Responses, Nagoya University Graduate School of Medicine, Tsurumai-cho 65, Showa-ku, Nagoya 466-8550, Japan

**Keywords:** ovarian cancer, plasma-activated medium, plasma-activated lactate Ringer’s solution, aqueous plasma, intraperitoneal metastasis, macrophage, immune response

## Abstract

**Simple Summary:**

Ovarian cancer is among the most malignant gynecologic cancers, in part because intraperitoneal recurrence occurs with high frequency due to occult metastasis. We have demonstrated a metastasis-inhibitory effect of plasma-activated medium (PAM) in ovarian cancer cells. Here, we investigated whether PAM inhibits intraperitoneal metastasis. We observed that PAM induced macrophages’ infiltration into the disseminated lesion, which was co-localized with inducible nitric oxide synthase (iNOS)-positive signal, indicating that PAM might induce M1-type macrophages. We also observed that intraperitoneal washing with plasma-activated lactate Ringer’s solution (PAL) significantly improved the overall survival rate in an ovarian cancer mouse model. Intraperitoneal washing therapy might be effective to improve clinical outcomes of ovarian cancer.

**Abstract:**

Epithelial ovarian cancer (EOC) is the most lethal gynecologic malignancy. The major cause of EOC’s lethality is that intraperitoneal recurrence occurs with high frequency due to occult metastasis. We had demonstrated that plasma-activated medium (PAM) exerts a metastasis-inhibitory effect on ovarian cancer in vitro and in vivo. Here we investigated how PAM inhibits intraperitoneal metastasis. We studied PAM’s inhibition of micro-dissemination onto the omentum by performing in vivo imaging in combination with a sequential histological analysis. The results revealed that PAM induced macrophage infiltration into the disseminated lesion. The iNOS-positive signal was co-localized at the macrophages in the existing lesion, indicating that PAM might induce M1-type macrophages. This may be another mechanism of the antitumor effect through a PAM-evoked immune response. Intraperitoneal lavage with plasma-activated lactate Ringer’s solution (PAL) significantly improved the overall survival rate in an ovarian cancer mouse model. Our results demonstrated the efficiency and practicality of aqueous plasma for clinical applications.

## 1. Introduction

Ovarian cancer is one of the most lethal gynecological malignant diseases. Annually, there are approx. 300,000 new cases of ovarian cancer and 180,000 deaths from it worldwide. It is the eighth leading cause of cancer-related death for women, and only 46% of patients survive >5 years after the diagnosis [1,2]. Almost 75% of patients with ovarian cancer are diagnosed at an advanced stage because of its asymptomatic progression, accompanied by transcoelomic metastasis in the peritoneal cavity [3]. The current treatment strategy for ovarian cancer is debulking surgery followed by a combination of paclitaxel + platinum chemotherapy. However, the prognosis of advanced or recurrent patients remains poor because due to refractoriness to the chemotherapeutic agents.

The peritoneal mesothelial tissue is the most frequent metastatic site of ovarian cancer (in addition to perilesional tissues and lymph nodes through the transcoelomic route), unlike most of the other epithelial malignant diseases. About 70% of patients with ovarian cancer have peritoneal metastases [4]. Intraperitoneal recurrence may therefore be unavoidable because not all of the disseminated tumor cells would be sensitized to chemotherapy. The control of intraperitoneal dissemination for ovarian cancer would thus be an important treatment strategy. Intraperitoneal delivery of chemotherapy combined with cytoreductive surgery has been demonstrated to improve prognosis by eliminating micro-metastasis more effectively than intravenous administration [5].

Non-equilibrium atmospheric pressure plasma (NEAPP) has been studied as a promising tool for clinical applications such as sterilization, wound healing, and cancer treatment [6,7,8,9]. Reactive oxygen and nitrogen species (RONS) generated by NEAPP are proposed as the main functional players for a physiological response. There are two different modalities for the medical application of NEAPP: direct exposure of NEAPP to tissues and cells or the administration of plasma-activated solutions, e.g., plasma-activated medium (PAM) and plasma-activated Ringer’s lactate solution (PAL). Plasma-activated solutions present many advantages, such as lower cost, easier application, relatively stable, and less injury. Especially plasma-activated solutions could be applied to a wide and closed area such as the body cavity. The significant antitumor potential of plasma-treated solutions including the above two solutions has been demonstrated in various types of cancers including brain, lung, breast, gastric, pancreatic, blood, mesothelial, and gynecologic cancers [10,11,12,13,14,15,16,17,18,19]. In particular, it might be more practical to treat disseminating cancer in the body cavity, which is a typical symptom of gastric, pancreatic, mesothelial, and ovarian cancer. We have demonstrated that the intraperitoneal administration of plasma-activated solution in a mouse model effectively suppresses the peritoneal dissemination of ovarian, gastric, and pancreatic cancer, suggesting possibilities for real-world applications [14,15,20]. For example, PAM exhibited a metastasis-inhibitory effect on ovarian cancer in the abdominal cavity by inhibiting cancer cells’ metastatic abilities such as invasion, migration, and implantation onto the peritoneum—especially the mesothelium, which is covered by a monolayer of mesothelial cells [20].

Here, we further investigated how PAM suppresses the microdissemination of cancer cells on the omentum in an ovarian cancer mouse model. Our findings indicated that M1-like macrophages, which are known as tumor-suppressing immune cells [21], are partially responsible for this suppressive effect. We also examined aqueous plasma washing therapy in which PAL is perfused intraperitoneally, and our results revealed its survival benefit compared to the controls. This therapy could be advantageous for the improvement of clinical outcomes for ovarian cancer.

## 2. Results

### 2.1. PAM Inhibits the Cell Viability of Various Types of Ovarian Cancer Cells

**Figure 1:**
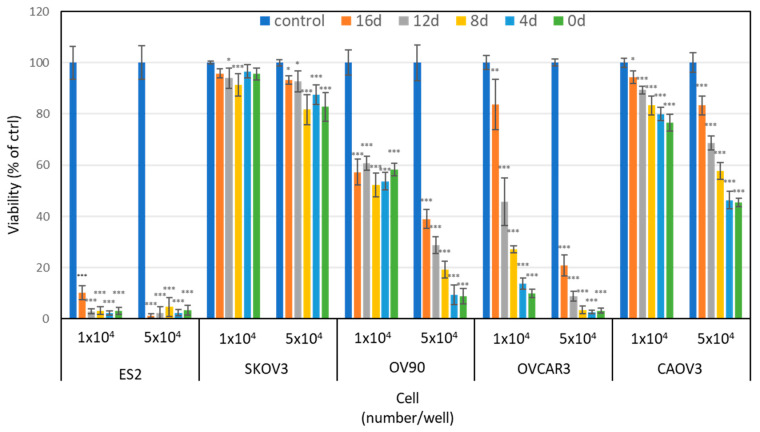
Antitumor effect of plasma-activated medium (PAM) on various ovarian cancer cells, depending on the cell type, cell number, and PAM storage periods. The antitumor effect of PAM was analyzed on day 0, 4, 8, 12, and 16 after the preparation. Two cell numbers (5 × 10^3^ and 1 × 10^4^ per well) were analyzed for the antitumor effect. The data are mean ± SD. Three independent experiments were performed. A one-way ANOVA with Tukey’s post hoc test for equal variances was carried out between control and each storage period of PAM treated cells. Statistics are shown as *** *p* < 0.001; ** *p* < 0.01; * *p* < 0.05.

We first carried out a cell viability assay to assess the anti-tumor effect of PAM in five ovarian cancer cell lines: ES2, SKOV3, OV90, OVCAR3, and CAOV3. Figure 1 shows the viability of the cells treated with different storage periods of PAM at two different cell numbers for 24 h: 0, 4, 8, 12, and 16 days. The antitumor effect of PAM decreased in four of the ovarian cancer cell lines in a storage period-dependent manner; the exception was for ES2 cells. In addition, the PAM treatment was more effective on lower cell numbers in all five cell lines. This result indicated that each cell line showed different sensitivity to PAM, suggesting that each cell line might have a different mechanism to induce the PAM-antiproliferative effect. We had previously used ES2 cells in an ovarian cancer mouse model because it is one of the most malignant ovarian cancer cell lines. Herein, we observed that ES2 was the most sensitive to PAM of all five cell lines. It would thus be better to use ES2 cells for the assessment of PAM’s antitumor effect. Our present results indicated that PAM had an antitumor potential in a broad range of ovarian cancer cells.

### 2.2. The Antitumor Effect of PAM Is Antagonized by Iron Chelator on Ovarian and Normal Cells

**Figure 2:**
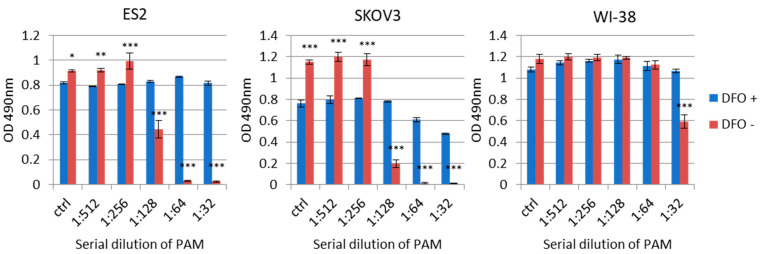
The influence of intracellular iron on the antitumor effect of PAM in cancer cells and normal cells. PAM was prepared with the same conditions as those used in the animal model. Cells were pre-treated with 200 μM DFO (Desferal for injection 500 mg, Novartis Pharma, Tokyo, Japan) before PAM treatment. ES2 and WL-38 cells were seeded at a density of 1 × 10^4^ cells, and SKOV3 cells were seeded at a density of 3 × 10^3^ cells in the wells of 96-well plates. Cell viability was assayed by a 3-(4,5-dimethylthiazol-2-yl)-5-(3-carboxymethoxyphenyl)-2-(4-sulfophenyl)-2H-tetrazolium, inner salt (MTS) assay at the corresponding PAM dilution ratio. Data are the mean ± SD. Three independent experiments were performed. The two-way ANOVA with Tukey’s post hoc test for equal variances was carried out between DFO-treated and -untreated cells. Statistics are shown as *** *p* < 0.001; ** *p* < 0.01; * *p* < 0.05.

It was reported that plasma-generated ROS, such as hydrogen peroxide (H_2_O_2_), are one of the key players for the antitumor effect of PAM. H_2_O_2_ in an aqueous solution is relatively stable and not highly reactive to biomolecules, but once it encounters iron or copper, it rapidly reacts to generate a hydroxyl radical (•OH), which may become the main cause of almost all of the induction of the antitumor effect. Therefore, to further investigate whether H_2_O_2_ is responsible for this effect, we used the iron chelator deferoxamine (DFO). ES2 and SKOV3 cells and WI-38 (normal human lung fibroblasts) were treated with gradient ratios of diluted PAM or PAM with DFO, and we performed an MTS assay to check these cells’ viability (Figure 2). We observed that most of the antitumor potential of PAM was inhibited by adding DFO in the PAM in all three cell lines, indicating that •OH from the Fenton reaction is the main contributor to the efficacy of PAM.

### 2.3. Intraperitoneal PAM Injection Suppresses Abdominal Micrometastasis in a Mouse Model

**Figure 3:**
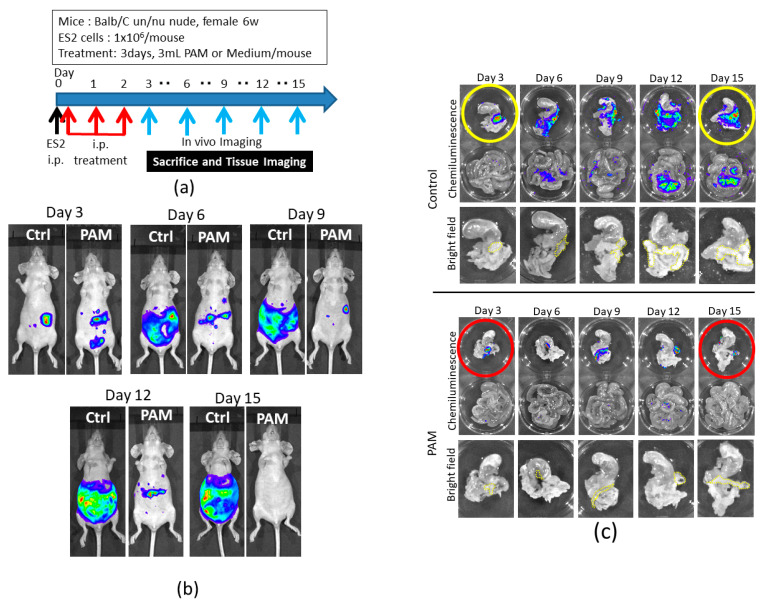
The antimetastatic effect of PAM in the ovarian cancer mouse model. (**a**) Outline of intraperitoneal PAM treatment in the model. (**b**) Peritoneal metastases in the PAM and control groups were evaluated every three days until day 15. (**c**) ES2 cells metastasized onto peritoneal tissues; omentum/pancreas with stomach were in the upper dish, mesenterium was in the lower dish, and their chemiluminescence was detected by the Xenogen IVIS 200 Imaging system. Yellow dashed lines in the bright field were indicated mouse omenta. The circled tissues were analyzed in the subsequent experiment.

We had demonstrated that PAM treatment significantly inhibited the intraperitoneal metastasis of ES2 cells and improved the survival rates compared to the control group. We then focused on the inhibitory effect of microdissemination at an early stage of cancer progression. A protocol of the animal experiment is shown in Figure 3a, which is the same as the previous experimental conditions [20]. Here, to examine the microdissemination on the omentum, we monitored the chemiluminescence from luciferase-expressing ES2 cells using in vivo imaging, i.e., the IVIS 200 Imaging System (Caliper Life Science, Hopkinton, MA, USA) every three days. The mice that showed the highest intensity signal in the PAM and control groups were sacrificed, and the peritoneal metastatic organs were assessed by chemiluminescence again to determine where ES2 cells disseminated on the organs including the omentum. As shown in Figure 3b, an intraperitoneal PAM injection markedly inhibited the progression of ovarian cancer cells in the abdominal cavity compared to the controls in which mice were treated with a non-plasma-irradiated medium. We also demonstrated that, in contrast to the controls, PAM significantly suppressed ES2 cells’ metastasis on the omentum as well as mesentery (Figure 3c). These results confirm those of our previous report [20]. Moreover, ES2 cells were on the omentum on day 3 in both groups, although after day 3, the cells sequentially proliferated only in the control group, suggesting that PAM may inhibit the proliferation of ES2 cells even after their seeding on the omentum.

To apply this new cancer treatment in a clinical setting, we should consider any side effect of PAM in the whole body. We analyzed the whole organs and blood in mice treated with PAM without ES2 cell injection on day 3, just after PAM treatment, and day 24, after three weeks of PAM treatment. As shown in Table 1, the concentration of lactate dehydrogenase (LDH) of the PAM-treated group on day 3 was higher than that of the control group, indicating that cells in the body might be abruptly affected by PAM. However, this elevation was recovered after three weeks of the treatment. Hypertrophy of the pancreas lymph nodes, an abdominal organ, was induced on day 3, and this was also restored on day 24 after the PAM treatment (Figure S1). Based on these results, we speculate that PAM might temporarily induce non-severe damage in the mouse body.

### 2.4. PAM Suppresses ES2 Cell Growth on Metastasized Omentum

**Figure 4:**
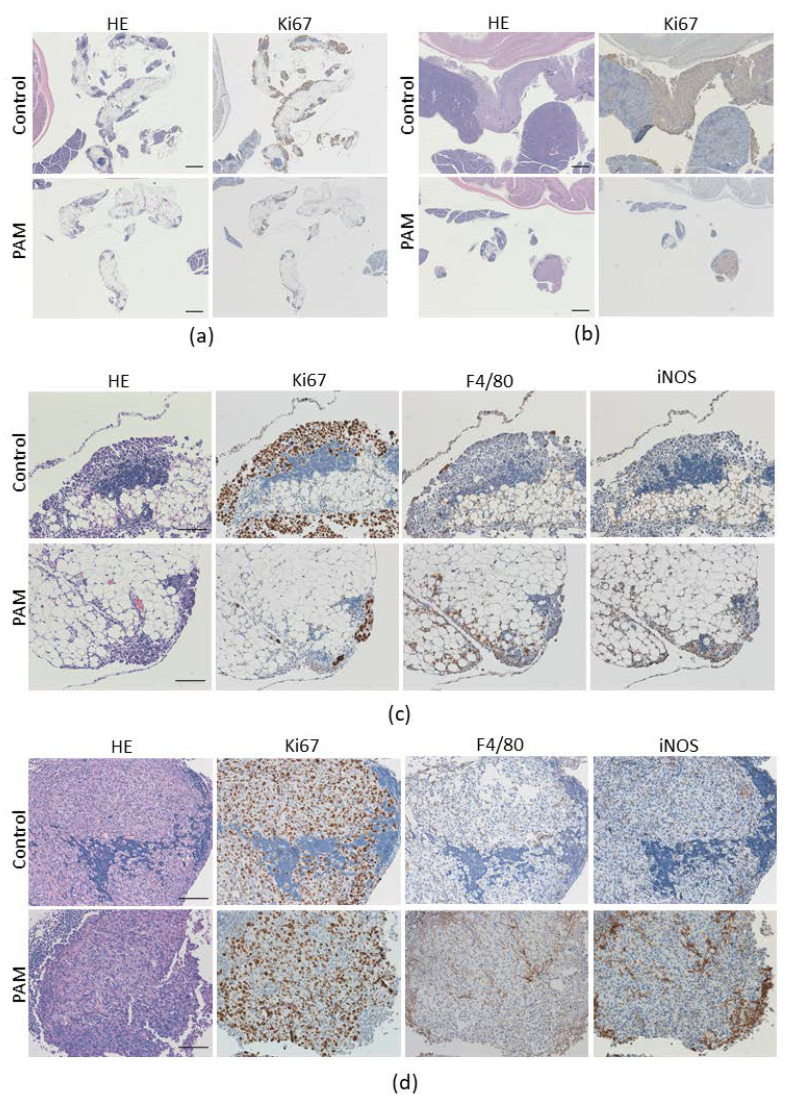
Antimetastatic and proliferative effect of PAM on mouse omentum tissue. The sections corresponding with circled tissues in Figure 3c were used for the indicated immunohistochemical staining as follows: (**a**) Hematoxylin-eosin (HE) and Ki-67 staining on day 3 and (**b**) day 15, (**c**) HE, Ki-67, F4/80 as a macrophage marker, and iNOS staining on day 3 and (**d**) day 15. The scale bars in (**a**,**b**) correspond to 500 nm and the scale bars in (**c**,**d**) correspond to 100 nm, in 25× and 200× magnification images, respectively.

To examine how PAM suppresses the growth of ES2 cells on the omentum, we performed a histological analysis on disseminated omentum lesions on day 3 and day 15 after an intraperitoneal injection of ES2 cells. As shown in Figure 4a, Ki67-positive cells, indicating proliferating cells such as cancer cells, were fewer in number on the surface of the omentum treated with PAM compared to the controls on day 3. The Ki67-positive lesions were enlarged even in the pancreas of the controls on day 15, whereas Ki67-positive cells in the PAM-treated tissue were confined in the omentum (Figure 4b). These findings are correlated with the results of the in vivo imaging at the late stage of cancer progression, but the differences between the PAM and control groups on day 3 were not reflected by the in vivo imaging results, in which obvious differences were not detected. These results demonstrate the PAM-inhibitory effect of ES2 cells’ microdissemination on the omentum.

We next investigated whether immune cells contribute to the anti-dissemination effect of PAM. Macrophages play an important role in the tumor microenvironment, regulating cancer progression. We thus analyzed tumor-infiltrating macrophages in a disseminated site of the omentum, and we observed that the number of infiltrating macrophages in the PAM-treated tissue was higher than in that of the control tissue at day 3 and day 15 after the injection of ES2 cells (Figure 4c,d). We also determined whether the infiltrating macrophages were colocalized with inducible nitric oxide synthase (iNOS), which is a marker of M1-like macrophages, and the results clearly demonstrated that the around 60% iNOS-positive signal was present and corresponded to F4/80-positive macrophages’ existing site in the PAM-treated tissue at days 3 and 15 (60.3 ± 15.2% and 63.8 ± 40.4% respectively; Figure 4c). There were minor amounts of iNOS signal in the controls (15.4 ± 6.9% at day 3 and 24.9 ± 5.4% at day 15; PAM vs. Ctrl at day 3 had statistical significance, *p* < 0.001; Figure 4d). These findings indicated that PAM might promote M1-type macrophage infiltration and eliminate the ES2 cancer cells in the disseminated site on the omentum via PAM’s immunologic adjuvant properties.

### 2.5. Intraperitoneal PAL Washing Therapy Prolongs the Overall Survival in an Ovarian Cancer Mouse Model

**Figure 5:**
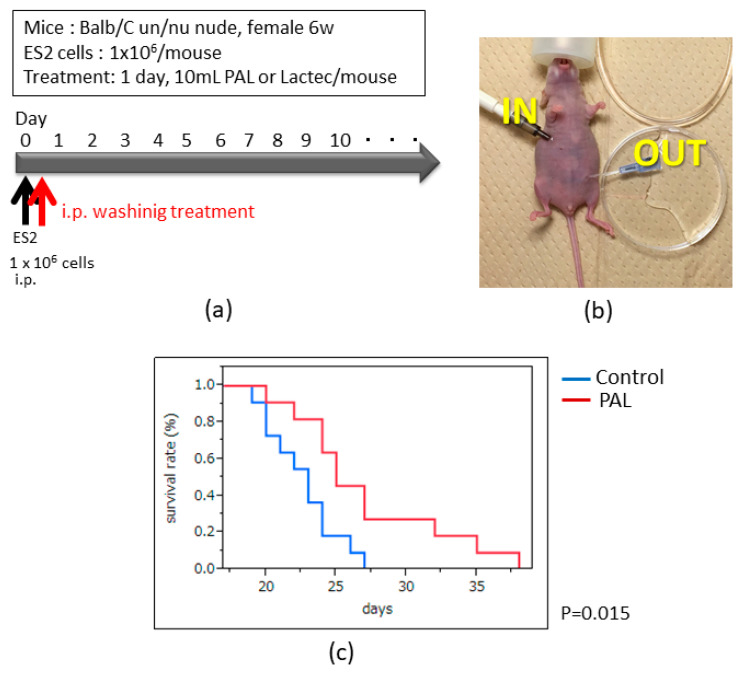
Effect of plasma-activated Ringer’s lactate solution (PAL) washing therapy on intraperitoneal metastasis in the ovarian cancer mouse model. (**a**) Outline of PAL washing therapy in the model. (**b**) The perfusion route during the therapy. (**c**) Survival analysis by the Kaplan–Meier method in the PAL washing and control groups (*p* < 0.05, *n* = 11).

We previously demonstrated that an intraperitoneal PAM injection significantly improved the survival rate in an ovarian cancer mouse model. This treatment could be beneficial for intraperitoneal microdissemination, which is experienced by patients suffering from a recurrence of ovarian cancer. We propose a new treatment protocol, as indicated in Figure 5a, using PAL as an intra-abdominal washing solution prepared by new plasma equipment developed for medical use. According to our previous report, PAL prepared with oxygen and nitrogen gases into an argon flow exhibited a greater antitumor potential compared to any other condition in ES2 cells [22]. In the present study we thus tested PAL prepared with these conditions, i.e., 80% argon, 10% oxygen, and 10% nitrogen. In this experiment, PAL was perfused once into the abdominal cavity of mice through an indwelling needle with a syringe pump (KDS-220, KD Scientific, Holliston, MA, USA) to control the infusion rate but not the withdrawal rate (Figure 5b). The results showed that the survival rates were significantly improved in the PAL washing group compared to the control group (Figure 5c, *p* < 0.05). This result indicated that the PAL washing therapy could be used in clinical practice in the future.

## 3. Discussion

Standard treatments for ovarian cancer have been developed such as cytoreductive surgery followed by platinum-taxane combination chemotherapy. Despite patients’ relatively high sensitivity to chemotherapy, the prognosis of advanced or recurrent ovarian cancer with intraperitoneal metastasis is poorer than without the metastasis, and it is, therefore, important to target peritoneal metastasis in ovarian cancer treatment. The peritoneal mesothelium in the abdominal cavity of the patient is a main site of metastasis. Once the ovarian cancer cells are released from the primary lesions, they are easily transported almost everywhere within the cavity through ascites and become attached to the mesothelium. The cases of 5% of patients diagnosed with early-stage epithelial ovarian carcinoma were complicated with omental metastasis even with no macroscopic tumor implant [23]. In this study, we demonstrated the antimetastatic potential of PAM in our animal model which may reflect a patient with Stage IC3 ovarian cancer, defined as a tumor limited to one or both ovaries with malignant cells in ascites or peritoneal washings [24]. This stage of ovarian cancer might be spread throughout the peritoneal cavity and form micrometastatic dissemination on the mesothelium.

Intraperitoneal chemotherapy is based on the same concept as intraperitoneal PAM treatment. Many clinical trials of this chemotherapy have been conducted, and their results demonstrated its clinical advantage compared to the intravenous method; however, patients were suffering from serious abdominal complications because of the chemotherapy’s severe toxicity and catheter-related problems [25,26]. We have observed the selective cytotoxicity of PAM in cancer cells [12,16,27] as well as the selective effect of PAL [22]. In the present study, we assessed the safety of intraperitoneal PAM treatment in detail and observed that there were a few symptoms such as an increased LDH level in the blood and pancreas lymph node hypertrophy in the acute phase; however, these were improved on day 24 after the treatment, suggesting that PAM treatment may not have severe toxic effects, unlike intraperitoneal chemotherapy. This preclinical safety evaluation was based on, in part, the ICH-S6(R1) guideline. Furthermore, a single dose of PAL-washing improved the survival without catheterization. We propose that PAL-washing therapy could be a practical option as adjuvant therapy after debulking surgery to improve the clinical outcomes of ovarian cancer.

The biological effect of indirect plasma (such as plasma-activated solutions) is mainly responsible for RONS, which are produced by plasma, a cluster of highly reactive components [28]. Several recent studies revealed that ROS generated by plasma might induce cell death through various alterations such as damage to DNA, the cell cycle, mitochondrial metabolism, signal transduction, and gene expression [29,30,31,32,33]. Only a few studies have reported the antimetastatic effect of direct plasma [34,35]. We have also demonstrated in a mouse model that PAM can suppress ovarian cancer cell migration and invasion and ROS, which is partially attributed to the antimetastatic potential of PAM [20]. PAM introduces excessive ROS into a cell and increases the intracellular ROS. Cells are highly regulated by many molecules to metabolize ROS, mainly in mitochondria. Our present findings revealed that each ovarian cancer cell line presented different susceptibility to PAM, suggesting that different ROS metabolites’ ability might be partially responsible for this different susceptibility.

Iron metabolism has one of the most important roles to regulate oxidative stress in cells, as iron directly reacts as a catalytic substance with ROS, and a more cytotoxic ROS, i.e., •OH was generated via the Fenton reaction [36,37]. Shi et al. demonstrated that the anti-proliferative effect of plasma could be potently controlled by the amount of iron in malignant mesothelioma cells [19]. We also investigated the anti-proliferative effect of PAM in ovarian cancer cells that were strongly inhibited by DFO, which uses as an agent for iron-overload disease. Intracellular catalytic ferrous iron may be a key factor to facilitate the anti-proliferative effect of PAM. Interestingly, DFO-treated ES2 cells showed a completely inhibited anti-proliferative effect even though the cells were relatively highly sensitive to PAM compared to other ovarian cancer cells, leading us to speculate that the death of ES2 cells treated with PAM was induced in an iron-dependent manner, and that PAM might induce ferroptosis [38,39].

Macrophages play an essential role in host defense as well as tissue remodeling through a variety of functions such as immunity, phagocytosis, cytotoxicity, and inflammation. Immature monocytes secreted from the bone marrow are circulated everywhere in the body via the bloodstream. Once they have migrated into tissues, they are differentiated into mature macrophages, where they change their physiological activity in response to environmental cues, enabled by their notable plasticity [21,40]. Macrophages as well as fibroblasts, adipocytes, and other hematopoietic cells are also recruited into a tumor to promote its progression and metastasis in the tumor microenvironment, and these cells are referred to as tumor-associated macrophages (TAMs). There are two major polarization states in macrophages: the classically activated M1 subtype, and the alternatively activated M2 subtype. The M1 macrophages are characterized by the secretion of pro-inflammatory cytokines which activate antitumor immunity. Conversely, M2 macrophages exert anti-inflammatory and pro-tumorigenic properties, similar to TAMs [41]. Several studies revealed that high levels of TAMs correlate with poor prognosis in many cancer types, and the blocking of macrophage infiltration into tumors and changing their polarization from the M2 to the M1 subtype could be a possible target for cancer treatment [42,43,44]. Recent reports have shown that plasma-induced immunogenic cancer cell death (ICD) can activate an antitumor immune response [45,46,47]. Moreover, Kaushik et al. demonstrated that plasma-stimulated macrophages suppress cancer progression through pro-inflammatory cytokines [48]. A very recent study by Lee et al. showed that plasma-treated nitric oxide containing water upregulated the anticancer potential by controlling macrophage polarization [49]. We also demonstrated that PAM might promote M1 macrophages’ infiltration into the cancer cells’ disseminated lesion on the omentum, leading to anti-proliferative and anti-metastatic properties. It should be noted that we used an immune-deficient nude mouse strain in which T cells are impaired for the ovarian metastatic model. To further investigate whether PAM induces regulatory T cells through activated M1 macrophages, a new syngeneic ovarian cancer mouse model should be developed. Further investigations are necessary to test and further elucidate the underlying mechanisms of a plasma-inducing immune response.

In the present study, we demonstrated the antimetastatic activity of PAM/PAL in the animal model which may reflect the early stage but not the advanced stage of ovarian cancer. A part of the results may help to predict its benefit for clinical treatment, such as intraabdominal washing treatment for the case with positive ascites cytology in early stage as well as the case with occult metastasis on the mesothelium in the advanced stage. To clarify the availability of PAM/PAL even in the advanced stage, further studies should be conducted using a suitable animal model that reproduces clinical situations to exploit the additional extension of clinical application.

## 4. Materials and Methods

### 4.1. Cell Cultures

The five human ovarian cancer cell lines ES2, SKOV3, OV90, OVCAR3, and CAOV3 and the normal human lung fibroblast cell line WI-38 were obtained from the American Type Culture Collection (ATCC; Manassas, VA, USA). These were maintained in RPMI-1640 medium (R8758, Sigma-Aldrich, St. Louis, MO, USA) supplemented with 10% heat-inactivated fetal bovine serum (FBS) and penicillin-streptomycin at 37 °C in a humidified atmosphere of 5% CO_2_. Stably expressed luciferase cells were generated using a recombinant retrovirus as described [50]. In brief, 293T cells were transfected with the pQCXIP vector encoding luciferase gene with the pVPack-GP and pVPack-Ampho vectors for the production of retrovirus particles (Agilent Technologies, Santa Clara, CA, USA). The culture supernatant of 293T cells was collected 48 h later and applied to ES2 cells with 2 mg/mL polybrene (Sigma-Aldrich). The cells were cultured for 24 h, and then 1 mg/mL puromycin (Sigma-Aldrich) was added for the selection of infected cells.

### 4.2. Plasma System and Preparation of PAM and PAL

We used two independent NEAPP-generating systems to prepare PAM and PAL. One is an open system, Habahiro, developed by Prof. M. Hori, Center for Low-temperature Plasma Sciences, Nagoya University, Nagoya, Japan [51,52], and the other is a closed system, Tough Plasma ((Fuji Corp., Aichi, Japan) in which the plasma head is covered with a chamber to eliminate air during the plasma irradiation to a solution, and the plasma flow can be of variable composition [22]. The details of each system have been described. RPMI medium without FBS was exposed to NEAPP generated by the Habahiro system excited by the application of 10 kV of a 60-Hz commercial power supply to two electrodes 8 mm apart under an argon (Ar) gas flow (2 L/min), with which we obtained PAM. Ringer’s lactate solution (Lactec Injection, Otsuka Pharmaceutical Co., Tokyo, Japan) was exposed to NEAPP by the Tough Plasma system by the application of 10 kV of a 60-mA/60-Hz AC power supply to 15-mm distant electrodes in an Ar gas flow (1.6 L/min) mixed with 0.2 L/min of oxygen and nitrogen gases (a total of 2 L/min) under purging with Ar gas in the chamber. The distance between the plasma source and the liquid surface (L) was fixed in each recipe. In the in vitro experiments, 10 mL of RPMI-1640 medium in a 60-mm dish (AGC Techno Glass Co., Shizuoka, Japan) was placed just below the plasma head (L = 25 mm) and exposed to plasma for 5 min. For the animal treatment, 5.5 mL of RPMI-1640 medium in a 12-well plate (Nunc, Thermo Fisher Scientific, Waltham, MA, USA) was placed with L = 3 and treated for 10 min [20]. For the PAL preparation in the animal experiment, 10 mL of lactate Ringer’s solution in a 60-mm dish was placed with L = 4 in the chamber, after purging with Ar gas, and exposed to plasma for 10 min [22].

### 4.3. Cell Viability Assay

An Aqueous One Solution Cell Proliferation Assay kit (Promega, Madison, WI, USA) was used to determine the cell viability according to the manufacturer’s instructions. Cells were plated in the wells of a 96-well plate, and on the following day the cells were treated with 100 μL of PAM for 24 h. Absorbance was measured at 490 nm with a microplate reader (ELx808; BioTek Instruments Japan, Tokyo, Japan).

### 4.4. Animal Study

All of the procedures involving mice and the experimental protocols were approved by the Animal Experimental Committee of the Graduate School of Medicine, Nagoya University (permission no. 20048). The animal study was carried out in accordance with the Guidelines for Animal Experiments of the Nagoya University School of Medicine. The details of the in vivo experimental procedure have been described [20]. Six-week-old female nude mice (BALB/C) were obtained from Charles River Laboratories Japan (Yokohama, Japan). A total of 1 × 10^6^ ES2 cells were suspended in 300 μL of phosphate-buffered saline (PBS) and intraperitoneally injected. PAM was administered after the cell injection on the same day and was subsequently administered 1×/day for two days after the first injection (*n* = 5). Non-plasma irradiated Medium was used as a control treatment (*n* = 5). Anesthetized mice were intraperitoneally injected with 75 mg/kg D-luciferin (Caliper Life Sciences, Hopkinton, MA, USA) to acquire images using the Xenogen IVIS 200 Imaging System (Caliper Life Sciences). After the mice were sacrificed, their intraperitoneal tissues were placed in the D-luciferin solution to obtain the images again by the same system.

### 4.5. Histological Analysis and Immunohistochemistry

Mice were sacrificed and tissues were harvested and fixed by 4% paraformaldehyde for the histological analysis. Paraffin sections were prepared at 4 μm thick and stained with hematoxylin and eosin (HE) to assess the morphological changes for the safety evaluation. The corresponding sections on separate slides were used for several immunohistochemical stainings. After blocking with 0.3% hydrogen peroxide/methanol and subsequent 5% normal serum, the sections were incubated with antibodies against Ki-67 (1:200; abcam, Cambridge, MA, USA), macrophages (1:50; Bio-Rad, Hercules, CA, USA), and iNOS (1:100; abcam). Immunohistochemical staining was visualized using an Vectastain Elite ABC kit (Vector Laboratories, Burlingame, CA, USA) following the manufacturer’s instructions. Counterstaining for the nucleus was performed with Mayer’s hematoxylin. The positive signal was measured using ImageJ/Fiji plugin software.

### 4.6. Intraperitoneal PAL Washing Therapy

The method of preparation of the ovarian cancer mouse model is the same as that described in Section 4.4. After ES2 cells were peritoneally injected, 10 mL of PAL was perfused into the intraperitoneal cavity with a 22G indwelling needle (Terumo Corp., Tokyo, Japan) placed on the upper side of the abdomen as shown in Figure 5b (*n* = 11). Another needle for effusion was placed in a diagonal position of the infusion needle. The infusion rate was controlled using a syringe pump (0.7 mL/min, KDS-220, KD Scientific), but the withdrawal rate was free. We conducted the same procedure in the control group with non-plasma treated Ringer’s lactate solution (*n* = 11). The survival analysis was performed using the Kaplan–Meier method.

### 4.7. Statistical Analysis

All data are expressed as the mean ± SD. Statistical differences were analyzed using the one-way ANOVA, the two-way ANOVA and the Student’s *t*-test. The Kaplan–Meier survival analysis was performed by JMP software (SAS Institute Japan, Tokyo, Japan). A *p*-value < 0.05 was considered to indicate statistical significance.

## 5. Conclusions

We demonstrated that the recruitment of M1-type macrophages in the tumor was in part responsible for the tumor-suppressive effect of PAM, indicating that PAM might affect the immune response toward the antitumor response in the progression of metastasis. Since the immune system in the body is highly controlled, this finding may open a window to the use of plasma therapy that can influence a host’s immunocompetence. To achieve successful plasma immune cancer therapy in clinical settings, we must investigate the effect of PAM on the whole immune system in tumorigenesis and reveal its underlying mechanisms.

The concept of PAL washing therapy might be suitable for the treatment of occult peritoneal metastasis, which causes intraperitoneal recurrence despite the performance of complete debulking surgery. Our present findings demonstrated the efficacy of PAL washing therapy in an ovarian metastasis mouse model. We speculate that aqueous plasma possesses the efficiency, practicality, and safety for clinical applications.

## Figures and Tables

**Table 1:** Biochemical test of mice treated with an intraperitoneal plasma-activated medium (PAM) injection.

Day		N	TP (g/dL)	ALB (g/dL)	T-Bil (mg/dL)	GLU (mg/dL)	TG (mg/dL)	T-cho (mg/dL)	BUN (mg/dL)	CRE (mg/dL)	Na (mEQ/L)	Cl (mEQ/L)	K (mEQ/L)	Ca (mg/dL)	GOT (IU/L)	GPT (IU/L)	LDH (IU/L)	ALP (IU/L)	γ-GTP (IU/L)
3	Ctrl	4	4.7 ±0.22	3.1 ±0.10	0.02 ±0.019	202 ±38.1	50 ±19.0	59 ±3.3	30.6 ±2.15	0.16 ±0.02	147 ±4.0	112 ±3.2	7.7 ±1.3	8.4 ±0.38	149 ±57.5	5 ±2.4	613 ±105.4	279 ±39.4	<3
	PAM	3	4.6 ±0.59	2.9 ±0.15	0.01 ±0.017	200 ±25.7	62 ±1.5	61 ±9.8	25.6 ±4.99	0.22 ±0.10	143 ±5.0	105 ±9.8	8.1 ±1.5	8.1 ±0.99	243 ±181.5	7 ±1.4	1187 ±957.4	294 ±16.8	<6
	*p* value		0.84	0.09	0.74	0.94	0.31	0.65	0.13	0.27	0.35	0.25	0.67	0.62	0.36	0.26	0.27	0.57	-
24	Ctrl	4	4.6 ±0.13	3.0 ±0.13	0.01 ±0.008	212 ±26.3	25 ±17.4	55 ±9.5	48.9 ±39.3	0.34 ±0.35	147 ±2.2	111 ±1.3	8.4 ±5.4	7.7 ±0.33	140 ±48.6	3 ±0.6	603 ±225.2	247 ±37.5	<3
	PAM	3	4.7 ±0.06	2.9 ±0.12	0.01 ±0.006	193 ±24.5	33 ±10.3	53 ±1.0	23.1 ±3.07	0.10 ±0.04	149 ±1.5	112 ±1.5	5.4 ±1.4	8.1 ±0.21	61 ±2.1	<3	216 ±95.2	218 ±23.4	<3
	*p* value		0.21	0.67	0.58	0.37	0.54	0.71	0.32	0.31	0.22	0.19	0.40	0.18	0.04 *	-	0.04 *	0.31	-
^1^ref. from charlse river		10	4.83 ±0.337	3.02 ±0.204	0.024 ±0.0117	147.1 ±23.44	19.1 ±6.77	78 ±16.02	27.8 ±10.32	0.183 ±0.0134	153.8 ±2.86	114.9 ±3.35	3.6 ±0.435	9.9 ±0.377	115.2 ±48.79	46.8 ±11.71	-	180.6 ±25.18	0

The outline of the PAM treatment was the same as that in Figure 3a without ES2 injection. ^1^ Reference from https://www.crj.co.jp/cms/crj/pdf/product/rm/information/balbcnu/Control_data_BALBc-nu_2010.pdf (24 November 2020). Data are the mean ± SD. The Student’s *t*-test was carried out between control (Ctrl) and PAM treated group (PAM). Statistics are shown as * *p* < 0.05. The abbreviations are as follows: TP; total protein, ALB; albumin, T-Bil; direct bilirubin, GLU; glucose, TG; triglyceride, T-cho; total cholesterol, BUN; blood urea nitrogen, CRE; creatinine, Na; sodium, Cl; chlorine, K; potassium, Ca; calcium, GOT; glutamate oxaloacetate transaminase, GPT; glutamate oxaloacetate transaminase, LDH; lactase dehydrogenase, ALP; alkaline phosphatase, γ-GTP; gamma-glutamyl transpeptidase.

## Data Availability

The datasets supporting the finding of this study are available within the article and its Supplementary Information, or from the corresponding author upon reasonable request.

## Supplementary Materials

The following are available online at https://www.mdpi.com/2072-6694/13/5/1141/s1, Figure S1. The effects of intraperitoneal PAM injection treatment in mice.
