# Supplementary material for: Preclinical Verification of the Efficacy and Safety of Aqueous Plasma for Ovarian Cancer Therapy
Source: Cancers (Basel). 2021 Mar 7;13(5):1141. doi: 10.3390/cancers13051141 (PMC7962102; doi:10.3390/cancers13051141)
Supplement: Supplementary file 1 [file cancers-13-01141-s001.pdf]

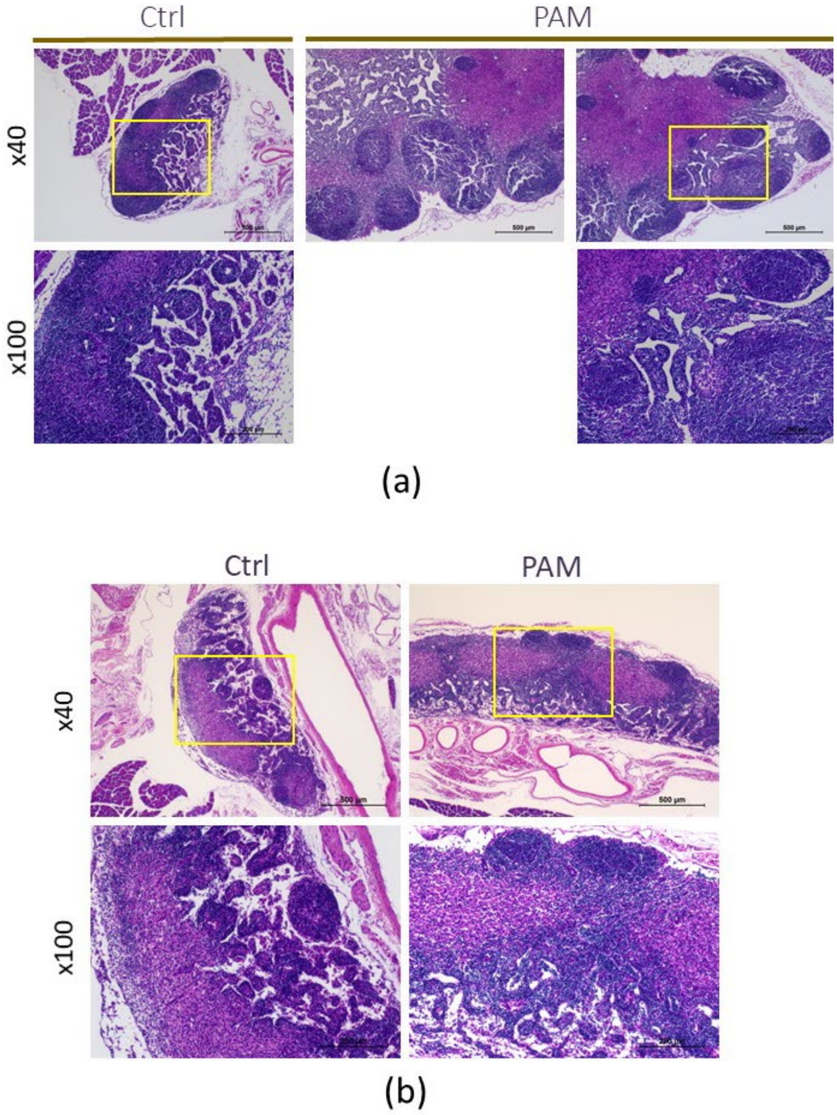

Figure S1. The effects of intraperitoneal PAM injection treatment in mice. The outline of the PAM treatment was the same as in Fig. 3(a) without ES2 injection. Paraffin sections were stained with HE and the morphological changes were assessed under microscopy. (**a**) Sections from day 3 after PAM treatment. (**b**) Sections from day 24 after PAM treatment. The scale bars correspond to 500 nm and 200 nm in 40×, 100× magnification images respectively.
